# Elicitors of Plant Immunity Triggered by Beneficial Bacteria

**DOI:** 10.3389/fpls.2020.594530

**Published:** 2020-11-09

**Authors:** Jelena Pršić, Marc Ongena

**Affiliations:** Microbial Processes and Interactions Laboratory, Terra Teaching and Research Center, Gembloux Agro-Bio Tech, University of Liège, Gembloux, Belgium

**Keywords:** plant immunity, systemic resistance, defense mechanism, molecular patterns, plant growth promoting rhizobacteria, biocontrol

## Abstract

The molecular basis of plant immunity triggered by microbial pathogens is being well-characterized as a complex sequential process leading to the activation of defense responses at the infection site, but which may also be systemically expressed in all organs, a phenomenon also known as systemic acquired resistance (SAR). Some plant-associated and beneficial bacteria are also able to stimulate their host to mount defenses against pathogen ingress via the phenotypically similar, induced systemic resistance phenomenon. Induced systemic resistance resembles SAR considering its mechanistic principle as it successively involves recognition at the plant cell surface, stimulation of early cellular immune-related events, systemic signaling via a fine-tuned hormonal cross-talk and activation of defense mechanisms. It thus represents an indirect but efficient mechanism by which beneficial bacteria with biocontrol potential improve the capacity of plants to restrict pathogen invasion. However, according to our current vision, induced systemic resistance is specific considering some molecular aspects underpinning these different steps. Here we overview the chemical diversity of compounds that have been identified as induced systemic resistance elicitors and thereby illustrating the diversity of plants species that are responsive as well as the range of pathogens that can be controlled via this phenomenon. We also point out the need for further investigations allowing better understanding how these elicitors are sensed by the host and the diversity and nature of the stimulated defense mechanisms.

## Introduction

Some bacteria isolated from the root microbiome have been selected for their remarkable beneficial effect provided to their host plant and are referred to as plant growth-promoting rhizobacteria (PGPR) (Backer et al., 2018; Singh et al., 2019). These PGPR favor plant growth notably by facilitating nutrient availability and modulating the host’s hormonal balance but also display plant protective activity toward pathogen ingress. This biocontrol potential relies on several traits including the ability to efficiently compete for space and nutrients with pathogens, a strong direct antagonistic activity based on secretion of low-size antimicrobials or hydrolytic enzymes and the capacity to stimulate induced systemic resistance (ISR) (Pieterse et al., 2014; Köhl et al., 2019). ISR is a systemically expressed resistance state that renders the host less susceptible to subsequent infection, and it is of great interest from an agronomical perspective because effective against a broad spectrum of microbial pathogens, nematodes, and insects (Pieterse et al., 2014; Grady et al., 2016; Rashid and Chung, 2017; Mhatre et al., 2018). Phenotypically, ISR resembles the systemic acquired resistance (SAR) mounted upon pathogen perception, which culminates from a complex immune-related process. At the front line, molecular features of invading microbes are detected with high specificity and sensitivity in the apoplast by a range of plasma membrane-anchored immune sensors referred to as pattern recognition receptors (PRR). These PRRs bind precise structural motifs (epitopes) of larger (macro)molecules retaining important functions for microbial fitness and thus widely conserved across species and termed microbe-associated molecular patterns (MAMPs). Some of the best described MAMPs from bacterial pathogens are flg22, a 22 amino acids portion of the flagellin protein, elf18 which is the epitope of the elongation factor EF-Tu, surface-exposed lipopolysaccharides (LipidA), and peptidoglycan (sugar backbone) (Schellenberger et al., 2019). The binding of MAMP by PRR initiates within minutes early immune-related events in responsive cells such as ion fluxes, oxidative burst, and phosphorylation cascade. Furthermore, defense mechanisms *sensu stricto* such as cell wall reinforcement and production of antimicrobial enzymes and secondary metabolites, referred to as phytoalexins, are stimulated (Piasecka et al., 2015). This PRR-mediated immune response [pattern triggered immunity (PTI)] is robust as it can detect MAMPs at nanomolar concentrations leading to fast and strong defensive responses (high transcriptional activation) but associated with growth-defense tradeoffs that reduce plant fitness (Huot et al., 2014). However, adapted pathogens use protein effectors injected into host cells to dampen PTI and subvert this first line of immune reaction. In turn, plants have evolved intracellular sensors called R (resistance) proteins, which interfere with these effectors leading to the effector-triggered type of immunity (ETI) which may, like PTI, lead to SAR (Kanyuka and Rudd, 2019; Schellenberger et al., 2019).

Due to the conserved nature of MAMPs, the plant’s receptor equipment would enable detection not only of pathogenic bacteria, but also of beneficials such as rhizobial symbiots and root-associated epiphytic PGPR (Zipfel and Oldroyd, 2017). These beneficial microbes have thus to evade (by hiding or changing their MAMPs) or suppress (by secreting effectors that interfere with immune responses and signaling) PTI in order to establish a mutualistic relationship with their host (Stringlis et al., 2018; Yu et al., 2019). This has been recently illustrated with the *Pseudomonas simiae* strain WCS417 first detected as a pathogen via its flg22 epitope, but which then strongly attenuates the host immune response presumably via T3SS-mediated delivery of effectors (Berendsen et al., 2015; Stringlis et al., 2019). Subversion of host immune reaction allows PGPR to sustainably colonize the rhizoplane and establish threshold populations necessary for providing beneficial functions including ISR triggering via the secretion of other elicitors not related to MAMPs.

Here we provide an updated overview of those PGPR determinants responsible for ISR elicitation that are in most cases small-size compounds secreted by the colonizing bacteria even if some proteins isolated from *Brevibacillus laterosporus*, *B. amyloliquefaciens*, and *Saccharothrix yanglingensis* have also been recently proposed as bacterial triggers (Table 1) (Wang et al., 2015, 2016; Zhang et al., 2018). We highlight their chemical diversity and structure- and dose-dependent activity but only refer to compounds that have been tested in pure (proven) form at biologically relevant concentrations and/or via specifically suppressed mutants and for which the ability to stimulate systemic resistance was clearly demonstrated to be independent from direct antimicrobial effect.

**TABLE 1:** PGPR produced elicitors of systemic resistance.

Elicitor	Strain	Plant	Pathogen	Method	References
**Acyl-homoserine lactones**					
Precise structure not defined	*Serratia liquefaciens* MG1	Tomato	*Alternaria alternata*	Mutant	Schuhegger et al., 2006
oxo-C14-HSL		Arabidopsis Barley	*P. syringae* pv. *tomato* DC3000 *Golovinomyces orontii Blumeria graminis* f. sp. *hordei*	Pure 6 μM	Schikora et al., 2011
		Arabidopsis	*P. syringae* pv *tomato* DC3000	Pure 6 μM	Schenk et al., 2012
	*Sinorhizobium meliloti* Rm2011	Arabidopsis	*P. syringae* pv *tomato*	Mutant	Zarkani et al., 2013
	*Sinorhizobium meliloti*	Barley Wheat Tomato	*Blumeria graminis* f. sp. *hordei Puccinia graminis* f. sp. *tritici Phytophthora infestans*	Mutant	Hernández-Reyes et al., 2014
**Cyclic lipopeptides**					
Surfactin	*Bacillus subtilis* 168	Bean Tomato	*Botrytis cinerea*	Mutant, pure 5 μM	Ongena et al., 2007
	*B. subtilis* UMAF6639	Melon	*Podosphaera fusca*	Mutant, Mutant + pure, pure 10 μM	García-Gutiérrez et al., 2013
	*B. amyloliquefaciens* S499/FZB42/QST713, *B. subtilis* 98S/BNO1, *Paenibacillus polymyxa* 56	Tomato	*B. cinerea*	SRF-producing strains, pure 10 μM	Cawoy et al., 2014
	*B. amyloliquefaciens* S13-3	Strawberry	*Colletotrichum gloeosporioides*	Pure 50 μM	Yamamoto et al., 2015
	*B. amyloliquefaciens* FZB42-AK3	*Lolium perenne* L.	*Magnaporthe oryzae*	Semi-purified _∼_250, _∼_500 μM	Rahman et al., 2015
	*B. subtilis*	Peanut	*Sclerotium rolfsii*	Pure 5, 10 μM	Rodríguez et al., 2018
	*B. amyloliquefaciens* S499	Wheat	*Zymoseptoria tritici*	Pure 1, 10, 100 μM	Le Mire et al., 2018
	*B. subtilis* BBG131/BBG125/Bs2504	*idem*	*idem*	Pure surfactin + mycosubtilin 100 μM, 50/50	Mejri et al., 2018
Mycosubtilin	*idem*	*idem*	*idem*	Pure _∼_100 μM	*same study*
Fengycin	*B. amyloliquefaciens* FZB42	Tomato	*S. sclerotiorum*	Pure _∼_60 μM	Farzand et al., 2019
Iturin	*B. amyloliquefaciens* S13-3	Strawberry	*Colletotrichum gloeosporioides*	Pure 10 μM	Yamamoto et al., 2015
	*B. amyloliquefaciens* 41B-1	Cotton	*Verticillium dahliae*	Pure 50 μM	Han et al., 2015
		Arabidopsis	*C. gloeosporioides*	Pure 10 μM	Kawagoe et al., 2015
	*B. vallismortis* EXTN-1	Chili pepper	*Phytophthora capsici*	Pure 1, 10 μM	Park et al., 2016
Massetolide A	*Pseudomonas fluorescens* SS101	Tomato	*P. infestans*	Mutant, pure _∼_ 45, 90 μM	Tran et al., 2007
Orfamide	*Pseudomonas* sp. CMR12a	*Brassica chinensis* Bean	*R. solani* AG 2-1 *R. solani* AG 4-HGI	Mutant	Olorunleke et al., 2015
	*Pseudomonas* sp. CMR12a	Bean	*R. solani* AG2-2	Mutant, pure 1–100 nM	Ma et al., 2016
	*Pseudomonas* sp. CMR12a	Rice	*Cochliobolus miyabeanus*	Mutant, pure 25 μM	Ma et al., 2017
Sessilin	*Pseudomonas* sp. CMR12a	Bean	*R. solani* AG 2-2 *R. solani* AG 4-HGI	Mutant	D’aes et al., 2011
	*Pseudomonas* sp. CMR12a	*Brassica chinensis* Bean	*R. solani* AG 2-1 *R. solani* AG 4-HGI	Mutant	Olorunleke et al., 2015
	*Pseudomonas* sp. CMR12a	Bean	*R. solani AG2-2*	Mutant, crude extract 1, 10 μg/L	Ma et al., 2016
Lokisin	*Pseudomonas* sp. COR10	Rice	*M. oryzae*	Crude extract 25 μg/ml, one CLP-producing strains	Omoboye et al., 2019
WLIP	*Pseudomonas* sp. COW10	*idem*	*idem*	*Idem*	*same study*
Entolysin	*Pseudomonas* sp. COR5	*idem*	*idem*	*Idem*	*same study*
**Rhamnolipids**					
	*P. aeruginosa*	Grapevine	*B. cinerea*	Pure _∼_150 μM	Varnier et al., 2009
	*P. aeruginosa*	Arabidopsis	*P. syringae* pv *tomato, Hyaloperonospora arabidopsidis, B. cinerea*	Pure _∼_300 μM	Sanchez et al., 2012
	*P. aeruginosa*	*Brassica napus*	*B. cinerea*	Pure 10, 100 μM	Monnier et al., 2018
***N-*alkylated benzylamine derivative**					
NABD	*P. putida* BTP1	Bean	*B. cinerea*	Mutant, pure 0.2 μM	Ongena et al., 2005
	*P. putida* BTP1	Bean Cucumber	*B. cinerea Colletotrichum lagenarium*	Pure 1 μM	Ongena et al., 2008
**Siderophores**					
Pyoverdine	*P. fluorescens* CHA0	Tobacco	*Tobacco necrosis virus* (TNV)	Mutant	Maurhofer et al., 1994
	*P. fluorescens* WCS374	Radish	*Fusarium oxysporum* f. sp. *raphani*	Mutant, pure 70 μg/root	Leeman et al., 1996
	*P. putida* WCS358	Tomato Bean Arabidopsis	*Colletotrichum lindemuthianum B. cinerea P. syringae* pv. *tomato*	Mutant, Pure 3.02 μg pyoverdine/g soil	Meziane et al., 2005
	*P. putida* WCS358r	*Eucalyptus urophylla*	*Ralstonia solanacearum*	Mutant, pure 10–100 μg/ml	Ran et al., 2005
	*P. fluorescens* WCS374r	Rice	*M. oryzae*	Mutant, pure 70 μg per root	De Vleesschauwer et al., 2008
Salicylic acid	*P. aeruginosa 7NSK2*	Bean	*B. cinerea*	Pure 100 nM	De Meyer et al., 1999
	*P. aeruginosa* 7NSK2	Grapevine	*B. cinerea*	Mutant	Verhagen et al., 2010
Pyochelin + SA/pyoverdine	*P. aeruginosa* 7NSK2	Tomato	*B. cinerea*	Mutant	Audenaert et al., 2002
**Elicitors with antibiotic function**					
DAPG	*P. fluorescens* CHA0	Tomato	*Meloidogyne javanica*	Mutant	Siddiqui and Shaukat, 2003
	*P. fluorescens* CHA0	Arabidopsis	*Peronospora parasitica*	Mutant, pure 10, 100 μM	Iavicoli et al., 2003
	*P. fluorescens* Q2-87	Arabidopsis	*P. syringae* pv. *tomato*	Mutant, pure 10–250 μM	Weller et al., 2012
		Arabidopsis	*P. syringae* pv. *tomato B. cinerea*	Pure 10, 100, 200 μM	Chae et al., 2020
Phenazine	*Pseudomonas* sp. CMR12a	Bean	*R. solani* AG 2-2 *R. solani* AG 4- HGI	Mutant	D’aes et al., 2011
	*Pseudomonas* sp. CMR12a	*Brassica chinensis* Bean	*R. solani* AG 2-1 *R. solani* AG 4-HGI.	Mutant	Olorunleke et al., 2015
	*Pseudomonas* sp. CMR12a	Rice Bean	*R. solani AG2-2*	Mutant pure 0.1, 1 μM	Ma et al., 2016
**Volatile organic compounds**					
2,3-butanediol	*B. subtilis* GB03, *B. amyloliquefaciens* IN937a	Arabidopsis	*Erwinia carotovora* subsp. *carotovora*	Mutant	Ryu et al., 2004
	*P. chlororaphis* O6	Tobacco	*E. carotovora*	Pure 100 μg/root	Han et al., 2006
		Pepper	*Cucumber mosaic virus*, *Tobacco mosaic virus*, *Pepper mottle virus*, *Tomato yellow leaf curl virus*, *Tomato spotted wilt virus*	Pure 1, 5, 10 mM	Kong et al., 2018
	*Enterobacter aerogenes*	Maize	*Setosphaeria turcica*	Pure 22 mM	D’Alessandro et al., 2014
3-pentanol	*B. amyloliquefaciens* IN937a	Pepper	*Xanthomonas axonopodis* pv. *vesicatoria*	Pure 10 μM, 1 mM	Choi et al., 2014
	*B. subtilis* GB03	Arabidopsis	*P. syringae* pv. *tomato* DC3000	Pure 100 nM, 10 μM	Song et al., 2015
Tridecane	*Paenibacillus polymyxa* E681	Arabidopsis	*Pseudomonas syringae* pv. *maculicola* ES4326	Pure 100 μM	Lee et al., 2012
Hexadecane	*Paenibacillus polymyxa* E681	Arabidopsis	*P. syringae* pv *maculicola*, *Pectobacterium carotovorum* subsp. *carotovorum*	Pure 1, 100 μM	Park et al., 2013

*Abbreviations: oxo-C14-HSL, *N,N*-dimethyl-*N*- tetradecyl-*N*-benzylammonium; NABD, *N,N-*dimethyl-*N*- tetradecyl-*N*-benzylammonium; DAPG, 2,4-diacetyl phloroglucinol.*

## PGPR Metabolites Identified as Elicitors of Plant Systemic Resistance

### Acyl-Homoserine Lactones

**FIGURE 1:**
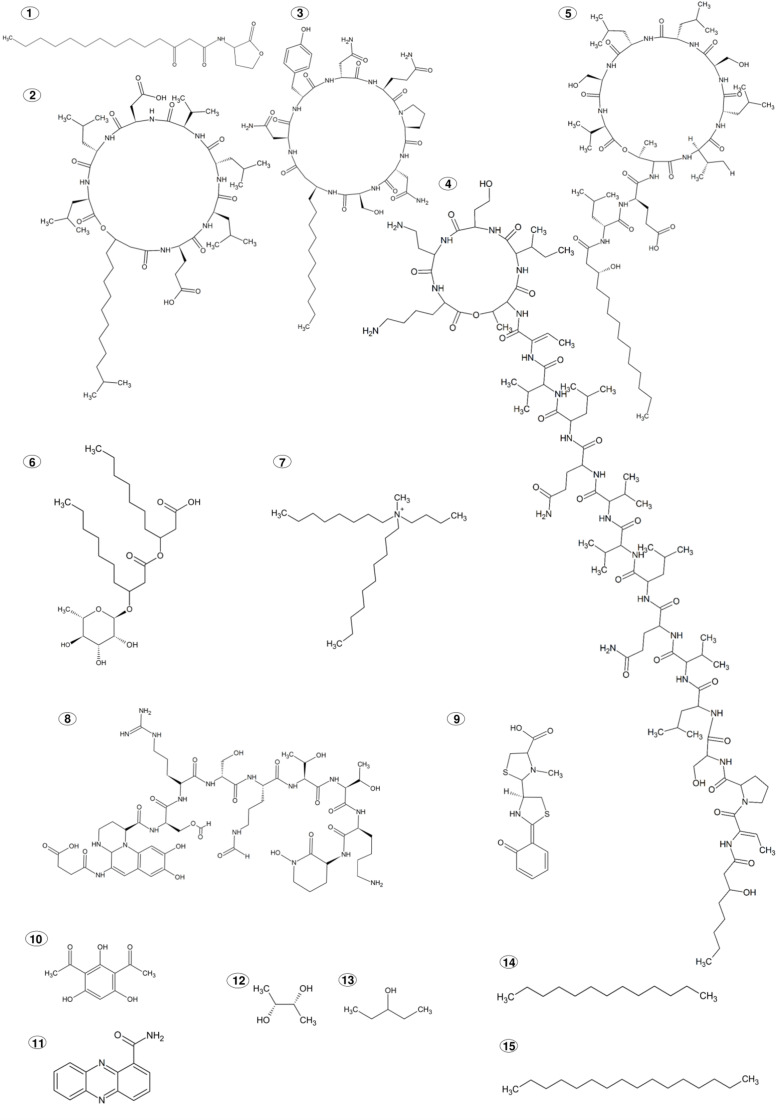
Chemical structures of PGPR produced ISR elicitors. **(1)** *N*-3-oxo-tetradecanoyl-L-homoserine lactone; **(2)** Surfactin; **(3)** Iturin; **(4)** Sessilin; **(5)** Orfamide; **(6)** Rhamnolipid, L-rhamnosyl-3-hydroxydecanoyl-3-hydroxydecanoic acid; **(7)** *N,N*-dimethyl-*N*- tetradecyl-*N*-benzylammonium; **(8)** Pyoverdine; **(9)** Pyochelin; **(10)** 2,4-diacetyl phloroglucinol; **(11)** phenazine-1-carboxamide; **(12)** 2,3-butanediol; **(13)** 3-pentanol; **(14)** Tridecane; **(15)** Hexadecane.

Many Gram-negative bacteria produce *N*-acyl-homoserine lactones (AHLs) as quorum-sensing molecules involved in cell-to-cell communication in order to monitor their behavior according to population density. Some AHLs are not only the mean of communication between bacterial cells, but also may be used as signal in inter-kingdom interaction and act as plant growth promoting compounds and/or as immunity elicitors (reviewed in Schikora et al., 2016). A first study showed, by using an AHL-suppressed mutant of *Serratia liquefaciens* MG1, that induced resistance in tomato toward *Alternaria alternata* by this strain is AHL-dependent (Schuhegger et al., 2006). Several works performed with purified molecules further revealed that AHL bioactivity is structure-dependent. AHLs with short length acyl chains have been mostly demonstrated to promote plant growth, whereas AHLs with longer fatty acid (C12, C14) are better described as elicitors of resistance (Schenk et al., 2012; Zarkani et al., 2013; Schikora et al., 2016). The long-chain *N*-3-oxo-tetradecanoyl-L-homoserine lactone (oxo-C14-HSL; Figure 1-1) has been amply demonstrated for immunity elicitation in many pathosystems. Upon treatment at 6 μM, oxo-C14-HSL successfully induced systemic resistance against biotrophs (*Blumeria graminis* f. sp. *hordei*, *Puccinia hordei*, *Golovinomyces orontii*) and hemibiotroph (*P. syringae* DC3000) in barley and Arabidopsis, but not against the necrotrophs *Botrytis cinerea* and *Plectosphaerella cucumerina* BMM (Schikora et al., 2011; Schenk et al., 2012; Wehner et al., 2019). ISR-eliciting activity of oxo-C14-HSL was also observed in wheat and tomato against *Puccinia graminis* f. sp. *tritici* and *Phytophthora infestans*, respectively (Hernández-Reyes et al., 2014). Collectively, these data indicate that the plant defensive response triggered by the same oxo-C14-HSL molecule may not be efficient in enhancing resistance against phytopathogens with necrotrophic lifestyle.

### Cyclic Lipopeptides

Rhizobacterial cyclic lipopeptides (CLPs) are multifunctional secondary metabolites involved in developmental processes such as motility and biofilm formation and in biocontrol primarily based on their antimicrobial activity (Ongena and Jacques, 2008; Geudens and Martins, 2018). However, some CLPs secreted by beneficial bacilli and pseudomonads have emerged as an important category of plant immunity elicitors as well. Surfactin is a heptapeptide occurring as a mix of naturally co-produced homologs varying in the length of the fatty acid chain (Figure 1-2). This CLP is among the *Bacillus* compounds best described as trigger of systemic resistance. When applied as pure compound on roots, in micromolar amounts (5–10 μM), surfactin demonstrated to induce ISR in bean, tomato, tobacco, against *B. cinerea*, in melon against *Podosphaera fusca*, and peanut, against *Sclerotium rolfsii* (Ongena et al., 2007; García-Gutiérrez et al., 2013; Cawoy et al., 2014; Rodríguez et al., 2018). The structure of this CLP may strongly impact its elicitor activity as observed on tobacco cells. Linear and/or methylated derivatives are much less efficient and only homologs with long C14 and C15 acyl chains are active at inducing early immune-related events, unlike C12 and C13 (Jourdan et al., 2009; Henry et al., 2011). Despite its high bioactivity on dicot plant roots, surfactin shows low competence in mounting immunity when applied on dicots leaves or monocots roots (Rahman et al., 2015; Yamamoto et al., 2015; Mejri et al., 2018). Induction of systemic resistance and/or plant defenses by other CLPs produced by biocontrol bacilli has also been occasionally reported. In tomato and grapevine, fengycin triggered ISR against *B. cinerea* and *Plasmopara viticola* (Farzand et al., 2019; Li et al., 2019), while CLPs from the iturin group (Figure 1-3) were shown to have a similar role in strawberry (Yamamoto et al., 2015), cotton (Han et al., 2015), grapevine (Farace et al., 2015) and Arabidopsis (Wu et al., 2018). Additionally illustrating the dependence of CLP-mediated ISR on the plant organ, iturin showed bioactivity on roots at concentration of 50 μM, whereas it was active already at 1–10 μM when applied on leaves (Han et al., 2015; Kawagoe et al., 2015; Yamamoto et al., 2015; Park et al., 2016). The activation of defense genes in Arabidopsis by iturin foliar treatment was also dependent on the molecule’s structure, i.e., cyclization and/or length of the b-hydroxy fatty acid chain (Kawagoe et al., 2015).

Several studies have also reported the involvement of *Pseudomonas* sp. CLPs in plant resistance stimulation on various pathosystems. Massetolide A was the first reported for its resistance-inducing activity in tomato against *P. infestans* (Tran et al., 2007). Recent works conducted with *Pseudomonas* sp. strain CMR12a revealed that two other types of CLPs, sessilin and orfamide (Figures 1-4,5, respectively) are involved in ISR-triggering albeit differently according to the pathosystem tested. Works combining the use of biosynthesis mutants and CLP extracts showed that both sessilin and orfamide are determinants of ISR in bean against *Rhizoctonia solani*, but that a balanced production is needed for optimal effect. These two compounds are indeed active at precise range of concentrations from 0.001 to 0.1 μM for pure orfamide, and 0.001 and 0.01 mg/L for sessilin as crude extract (Olorunleke et al., 2015; Ma et al., 2016). In monocots, such as rice, *Pseudomonas* CLP structure is crucial in determining elicitor effect on a given pathosystem. For example, orfamide applied at 25 μM was identified as elicitor inducing resistance to *Cochliobolus miyabeanus*, but it is not active against *Magnaporthe oryzae* (Ma et al., 2017). Nevertheless, CLPs such as WLIP, lokisin, and entolysin were recently described to successfully induce resistance toward this last pathogen, confirming elicitor specificity for certain pathosystems (Omoboye et al., 2019).

### Rhamnolipids

Rhamnolipids (RLs) are amphiphilic glycolipids produced by various species including pathogenic isolates, but also some plant beneficial *Pseudomonas* and *Burkholderia* species (Perneel et al., 2008; Abdel-Mawgoud et al., 2010). They are essential for bacterial surface motility and biofilm development (Vatsa et al., 2010; Chrzanowski et al., 2012). Mono- and di-RLs (Figure 1-6) tested as pure compounds have been shown to elicit plant defense responses and to induce resistance against various pathogens in grapevine, Arabidopsis, and *Brassica napus* using a wide range of concentrations from approximately 10 up to 300 μM (Varnier et al., 2009; Sanchez et al., 2012; Monnier et al., 2018, 2020).

### *N*-Alkylated Benzylamine Derivative

Although its function for bacterial life is not clear, an *N*-alkylated benzylamine derivative (NABD; Figure 1-7) produced by *P. putida* BTP1, was identified as elicitor (Ongena et al., 2005). Treatment of bean and cucumber roots with pure NABD at low micromolar concentration elicited similar protective effect compared to living cells. On the other hand, in tomato, the pure elicitor induced a lower protective effect than observed with the producing strain, suggesting the synthesis of an additional ISR determinant in that case (Ongena et al., 2008).

### Siderophores

To ensure their growth in iron-limited environments, microorganisms have evolved powerful Fe^3+^-acquisition systems based on the secretion of high-affinity iron-chelating molecules termed siderophores. PGPR siderophores are also known to antagonize pathogen populations by decreasing iron amounts in soil, resulting in competition for this essential element (Kramer et al., 2020). However, some siderophores also act as plant immunity elicitors and pyoverdines (also referred to as pseudobactines; Figure 1-8) produced by various fluorescent pseudomonads were, in the 90’s, among the first PGPR metabolites proposed as ISR elicitors. Their key role in systemic resistance induction was notably shown for *P. fluorescens* CHA0 on tobacco infected by *Tobacco necrosis virus*, and in the protection provided by *P. fluorescens* WCS374 to radish against Fusarium wilt (Maurhofer et al., 1994; Leeman et al., 1996). These chromopeptides were further described as inducers of resistance in various plant species such as bean, tomato, Arabidopsis, tobacco, eucalyptus and rice against a range of microbial pathogens (Meziane et al., 2005; Ran et al., 2005; De Vleesschauwer et al., 2008). In addition to pyoverdine, *P. aeruginosa* 7NSK2 also forms the chelating agent pyochelin (Figure 1-9) and its precursor salicylic acid (SA). When tested on pathosystem grapevine/*B. cinerea*, SA produced by *P. aeruginosa* 7NSK2 was crucial for mounting the plant immunity (De Meyer et al., 1999; Verhagen et al., 2010). However, for ISR stimulated in tomato by the same strain, an important role for SA could not be excluded, but probably combined with the action of other metabolites including pyochelin which may somehow also retain some eliciting activity (Audenaert et al., 2002).

### Elicitors With Antibiotic Function

To ensure fitness in the competitive rhizosphere niche, PGPR produce a wide range of secondary metabolites best identified for their antimicrobial function such as non-ribosomal peptides, polyketides, bacteriocins, terpenes, phenazines, quinolones, or rhamnolipids (Raaijmakers and Mazzola, 2012; Zhao and Kuipers, 2016; Tracanna et al., 2017). Interestingly, some of these antibiotics were also shown to act as signal for ISR stimulation at similar concentrations, making them promising tools for biocontrol with dual action on pathogen populations (Kenawy et al., 2019). One such antibiotic 2,4-diacetyl phloroglucinol (Figure 1-10), formed by *P. fluorescens*, is triggering resistance in Arabidopsis against various pathogens, such as *Peronospora parasitica*, *P. syringae* pv. *tomato*, and *B. cinerea*, upon application at relatively high 10–100 μM concentrations (Iavicoli et al., 2003; Weller et al., 2012; Chae et al., 2020). 2,4-diacetyl phloroglucinol can also induce resistance against nematodes as shown with *P. protegens* CHA0 for the reduction of infection caused by *Meloidogyne javanica* on tomato roots (Siddiqui and Shaukat, 2003). Besides, other *Pseudomonas* antibiotics of the phenazine-type were also reported to induce resistance. Notably phenazine-1-carboxamide (Figure 1-11) in rice toward *M. oryzae* at 0.1–1 μM, in bean toward *R. solani*, and pyocyanin produced by *P. aeruginosa* 7NSK2 in the pathosystem tomato/*B. cinerea* (Audenaert et al., 2002; D’aes et al., 2011; Ma et al., 2016).

### Volatile Organic Compounds

Most of the well-characterized PGPR elicitors are soluble compounds, but some volatile organic compounds (VOCs) were as well-reported to induce systemic resistance in the host plant, showing that these metabolites can also act as infochemicals involved in inter-kingdom communication (Kai et al., 2016). The most studied VOC immunity elicitor is 2,3-butanediol (2,3-BD; Figure 1-12) produced from glucose in the central metabolism (Yang et al., 2013). Its bioactivity was first assessed on Arabidopsis by application on roots, where *B. subtilis* GB03 induced resistance against *Erwinia carotovora* subsp. *carotovora*, while mutants deprived in 2,3-BD or its precursor acetoin production were inactive (Ryu et al., 2004). Bioactivity, when treated on roots in relatively high mM concentrations, was demonstrated as well in the pathosystems maize/*Setosphaeria turcica*, tobacco/*Erwinia carotovora*, and in pepper against multiple viruses (Table 1), where the immunity eliciting ability was structure-dependent. Namely, among the three forms, 2R,3R-BD (R), 2S,3S-BD (S) and 2R,3S-BD (meso), the S form is the least active (Han et al., 2006; D’Alessandro et al., 2014; Kong et al., 2018). However, on pepper against *Xanthomonas axonopodis* pv. *vesicatoria*, another VOC 3-pentanol (10 μM, 1 mM; Figure 1-13) showed higher activity than 2,3-BD (Choi et al., 2014). Beside 3-pentanol, long-chain VOCs tridecane and hexadecane (Figures 1-14,15, respectively) showed bioactivity as well at μM (100) concentration (Lee et al., 2012; Park et al., 2013).

## Molecular Basis of Plant Immunization: PGPR vs. MAMP Elicitors

The potential of PGPR to induce plant immunity mainly relies on the secretion of a range of structurally diverse low-molecular weight metabolites. However, the molecular mechanisms driving recognition of these elicitors at the plant cell surface are poorly understood. By contrast with MAMPs from pathogens, there is no indication so far for specific PRRs involved in the perception of PGPR elicitors. They activate immune responses only at relatively high μM concentrations compared to MAMPs, suggesting that they are not sensed via high-affinity receptors. CLPs are known to promptly insert into biological membranes causing defects, pore formation and cell lysis in a range of (micro)organisms (Balleza et al., 2019). Furthermore, immune response triggered by the *Bacillus* CLP surfactin was fully conserved in protease-treated tobacco cells, and successive applications do not lead to some refractory state due to the saturation of high-affinity binding sites (as receptors). Based on these and the fact that surfactin readily interact with sphingolipid-enriched microdomains in the plasma membrane, it was suggested that this CLP is perceived by plant cells via a lipid-mediated process. This is supported by the strong structure-dependent activity showing that only long fatty acid chain homologs are active at triggering early immune-related events in tobacco cells because they should display stronger interaction with lipid bilayers (Jourdan et al., 2009; Henry et al., 2011). Rhamnolipids also readily fit into plant lipid-based bilayer models. Therefore, it was proposed that by inserting into plasma membranes, these compounds provoke subtle changes in lipid dynamics that could be related to plant defense induction (Davis et al., 2010; Monnier et al., 2018; Schellenberger et al., 2019). As other alkyl-chain containing elicitors, AHLs could also be perceived by plant cells via a receptor-independent but lipid-mediated process, by analogy with data recently obtained on mammalian cells (Schikora et al., 2016; Song et al., 2018). It may explain why a given compound does not act at the same level on different targets, considering that the lipid composition in the plasma membrane differs according to the plant species/organs.

The way PGPR elicitors are perceived at the plasma membrane level is not clear, but there is still a clear convergence between PGPR-triggered immunity and pathogen-induced PTI in the early steps of downstream signaling. The best studied PGPR elicitors induce similar early immune events as observed upon pathogen perception, such as oxidative burst, ion fluxes, and phosphorylation cascade (Jourdan et al., 2009; Schikora et al., 2011; Cho et al., 2013; Cawoy et al., 2014; Farace et al., 2015; Rahman et al., 2015). Detection of MAMPs from pathogens leads to a fast and strong defensive response, but also to a costly reduction of the plant growth and fitness (Huot et al., 2014). On the other hand, PGPR and/or their elicitors usually prime the host to stimulate defense mechanisms but only after pathogen challenge as observed upon treatment with surfactin, AHLs, or with bacteria producing NADB or pyoverdines (De Vleesschauwer et al., 2008; Mariutto et al., 2011; Cawoy et al., 2014; Debois et al., 2015; Schikora et al., 2016). Again by contrast with PTI, PGPR elicitor-priming is not associated with major transcriptional reprogramming until the pathogen is detected and does not involve fitness costs but still prepares the plant for mounting a robust defense (Martinez-Medina et al., 2016; Mauch-Mani et al., 2017). Globally, the defense mechanisms restricting pathogen ingress that are stimulated by beneficial bacteria or their elicitors resemble those observed upon pathogen MAMP perception. It notably means up-regulation of genes involved in the synthesis of antimicrobial enzymes/proteins or metabolites and in hormone signaling. Higher expression of genes such are pathogenesis- related, lipoxygenase, plant defensin factor, and phenylalanine ammonia lyase, is often reported in elicitor pretreated plants after pathogen infection (Ongena et al., 2007; García-Gutiérrez et al., 2013; Zarkani et al., 2013; Song et al., 2015; Park et al., 2016; Yi et al., 2016; Kong et al., 2018; Song et al., 2019). Besides, PGPR elicitors also trigger stomatal closure and enhance cell wall reinforcement which serves as a structural barrier to pathogen invasion via callose deposition or accumulation of phenolic compounds and lignin (De Vleesschauwer et al., 2006; García-Gutiérrez et al., 2013; Schenk et al., 2014; Rodríguez et al., 2018).

## Discussion

From an agronomic perspective, ISR triggered by PGPR is interesting since the phenomenon is considered to provide long-lasting and broad-spectrum protection without causing growth cost and is not *a priori* conducive for development of resistance in pathogens (Köhl et al., 2019). However, the success of PGPR as stimulators of plant defenses is so far rather limited due to a range of factors. These include our global lack of knowledge about the nature and mode of action of their elicitors. Indeed, ISR determinants were identified mainly from a limited number of species (*Pseudomonas* spp. and *Bacillus* spp.), leaving elicitors from many other PGPR to be discovered. Moreover, in the last decades, tremendous advances have been done on understanding the basics of MAMP perception during PTI, but the mechanistic of PGPR elicitor recognition at the plant plasma membrane level and the molecular events underlying PGPR-induced priming remain largely obscure. As the lipid phase is suspected to act as docking platform for some of these elicitors, experimental biophysics and *in silico* dynamic modeling using appropriate biomimetic vesicles represent interesting approaches to get further insights into the physico-chemical basis of the interactions (Deleu et al., 2014; Balleza et al., 2019; Nishimura and Matsumori, 2020). It would help to explain why some compounds are only efficient on specific plants/tissues according to the nature and proportions of lipids in their domain-structured plasma membranes (Gronnier et al., 2018). Also, the variety of pathosystems tested so far is still limited and additional research on agriculturally important crops and pathogens is needed in order to better appreciate their potential at a larger market scale.

Furthermore, PGPR elicitors in most instances are active at micromolar doses but only a few studies suggest that quantities produced by bacteria *in planta* are sufficient to locally reach such threshold around the roots (D’Alessandro et al., 2014; Debois et al., 2015). Determining the amounts of elicitors produced by PGPR under natural conditions is still important, but not an easy task. It would require optimal extraction from rhizosphere samples and the most-advanced MS-based metabolomics allowing high sensitivity for their detection and quantification. Also, environmental factors may affect ISR efficiency (Williams et al., 2018), but their impact on the production of elicitors by PGPR remains poorly appreciated. Low temperature, acidic pH or poor oxygen availability are among the rhizosphere specific abiotic parameters that affect bacterial physiology and which may also modulate the production of secondary metabolites including elicitors as reported for *Bacillus* lipopeptides (Fahim et al., 2012; Pertot et al., 2013; Zhao and Kuipers, 2016). Biotic factors such as interactions with other microbial species of the soil microbiome or chemical cross-talk with the host plant, may also influence elicitor production under natural conditions (Debois et al., 2015; Wu et al., 2015; Venturi and Keel, 2016; Andrić et al., 2020). A better evaluation of the impact of all these factors deserve further investigation and is necessary to better anticipate inconsistencies in PGPR efficacy observed upon application under field conditions.

Integrating all this missing information should also lead to more rational determine the range of pathosystems, and environmental conditions in which PGPR-based bioproducts would be the most efficient. An alternative is to make from these bacterial immunogenic compounds microbial-derived products for the phytosanitary market provided that they can be produced and formulated in cost-effective industrial processes. This is feasible for some metabolites with high production rate in bioreactors, such as *Bacillus* lipopeptides (Zanotto et al., 2019; Brück et al., 2020), but the dose and structure dependent activity should guide a rational selection of the strain, optimization of culture conditions and extensive testing in field experiments in comparison with chemical products.

## Author Contributions

Both authors conceived the idea, designed the outlines of the review, and wrote the manuscript.

## Conflict of Interest

The authors declare that the research was conducted in the absence of any commercial or financial relationships that could be construed as a potential conflict of interest.
